# Bioactive Coatings on Titanium: A Review on Hydroxylation, Self-Assembled Monolayers (SAMs) and Surface Modification Strategies

**DOI:** 10.3390/polym14010165

**Published:** 2021-12-31

**Authors:** Julia Sánchez-Bodón, Jon Andrade del Olmo, Jose María Alonso, Isabel Moreno-Benítez, José Luis Vilas-Vilela, Leyre Pérez-Álvarez

**Affiliations:** 1 Grupo de Química Macromolecular (LABQUIMAC), Departamento de Química Física, Facultad de Ciencia y Tecnología, Universidad del País Vasco UPV/EHU, 48940 Leioa, Spain; Julia.sanchez@ehu.eus (J.S.-B.); jandrade@imasmed.com (J.A.d.O.); mariaisabel.morno@ehu.es (I.M.-B.); joseluis.vilas@ehu.es (J.L.V.-V.); 2 i+Med S. Coop, Parque Tecnológico de Alava, Albert Einstein 15, Nave 15, 01510 Vitoria-Gasteiz, Spain; jalonso@imasmed.com; 3 BCMaterials, Basque Center for Materials, Applications and Nanostructures, UPV/EHU Science Park, 48940 Leioa, Spain

**Keywords:** titanium, surface modification, bioactive coatings, pre-activation treatments, self-assembled monolayer (SAM), active layer, immobilization, controlled release

## Abstract

Titanium (Ti) and its alloys have been demonstrated over the last decades to play an important role as inert materials in the field of orthopedic and dental implants. Nevertheless, with the widespread use of Ti, implant-associated rejection issues have arisen. To overcome these problems, antibacterial properties, fast and adequate osseointegration and long-term stability are essential features. Indeed, surface modification is currently presented as a versatile strategy for developing Ti coatings with all these challenging requirements and achieve a successful performance of the implant. Numerous approaches have been investigated to obtain stable and well-organized Ti coatings that promote the tailoring of surface chemical functionalization regardless of the geometry and shape of the implant. However, among all the approaches available in the literature to functionalize the Ti surface, a promising strategy is the combination of surface pre-activation treatments typically followed by the development of intermediate anchoring layers (self-assembled monolayers, SAMs) that serve as the supporting linkage of a final active layer. Therefore, this paper aims to review the latest approaches in the biomedical area to obtain bioactive coatings onto Ti surfaces with a special focus on (i) the most employed methods for Ti surface hydroxylation, (ii) SAMs-mediated active coatings development, and (iii) the latest advances in active agent immobilization and polymeric coatings for controlled release on Ti surfaces.

## 1. Introduction

In the last decades, biomedical materials that are able to keep in contact with human cells for prolonged periods have been extensively developed for a wide range of applications related to human healthcare. These biomedical devices are widely used as non-implanted devices such as orthopedic fixation screws and as implants, sutures, catheters, dental and cardiovascular implants such as tents, heart valves or vascular grafts [1]. The selection of the material is crucial because it must meet a wide range of demanding properties. It must have high durability, appropriate interaction with tissue cells, no immunological or allergic response, and mechanical properties similar to those of human bone and corrosion resistance, among others. According to these requirements, the most commonly used materials in the manufacture of this type of device are polymers, ceramics or metals and their alloys. Among metals, titanium (Ti) and its alloys present excellent mechanical properties (good strength and low elastic modulus), biocompatibility, a great corrosion resistance caused by a stable oxide layer, good strength, and low density [2,3]. Due to these excellent characteristics, Ti has become the metal of choice in the fabrication of many of these biomaterials. In this context, Ti is usually employed either in its pure form or as an alloy with other metals such as vanadium, aluminum, tantalum, nickel or zirconium [4]. According to the American Society for Testing and Materials (ASTM) standards, commercially pure Ti (cpTi) has four different grades based on the amount of oxygen, nitrogen, hydrogen, iron and carbon generated during the purification procedures [5]. Among its alloys, Ti6Al4V and nickel-Ti are the mainly employed materials. Recently, the nickel–Ti alloy has gained attention because of its shape memory feature, which makes it suitable for self-expanding stents [6]. Compared to other alloys typically used in the biomedical field like cobalt-chromium-molybdenum (CoCrMo), Ti shows an absence of tissue toxicity and no allergic reaction [7,8].

Nevertheless, the osseointegration ability of these materials remains a challenge, since it depends on the primary stability and anchor effect of the implant, more than on the degree of contact [9]. The osseointegration process was first described 50 years ago by Bränemark as “a direct structural and functional connection between ordered, living bone and the surface of a load-carrying implant” [10]. Bränemark determined that, during effective implantation, a Ti oxide coating must anchor directly to living bone generating a linkage so strong that it is impossible to separate them without fracturing [10]. Nonetheless, according to the current literature, osseointegration is a complex and still unknown process, which depends on the immune system and the autonomic nervous system [11,12]. Therefore, in order to reduce the failure of early implants or to minimize complications in the healing process, principally for patients suffering from metabolic diseases, such as diabetes, osteoporosis and weakened immunity [13], Ti surface properties must be modulated to promote bone growth and expedite the apposition of new bone in the early stages after implantation [14]. Consequently, the regulation of implant surface in the early stages of cell attachment would allow a rapid and stable implantation, which would improve its short-term, and, more importantly, its long-term performance [15,16].

Infections are another concerning issue often associated with metal prosthesis implantation [17]. Unfortunately, Ti and its alloys are not biologically active by themselves and cannot avoid bacteria adhesion and proliferation, which can lead to infections that may even cause prosthesis rejection [18]. Accordingly, different approaches have been investigated to render Ti surfaces with antibacterial properties [19]. Most of them are based on the development of active coatings that usually imply surface modification with antibiotics, antimicrobial peptides or polymers or inorganic elements that provide antibacterial activity [20].

Hence, multifunctional Ti implants with good anti-infective ability, biocompatibility and even osteogenic ability are highly desirable. Thus, in the last years, different research groups have described different techniques such as laser treatment, anodization, and hydrothermal treatment, to modify and pre-activate Ti surfaces in order to improve their biocompatibility at both micro- and nano-scales [21,22]. Moreover, incorporation of active compounds through chemical modifications onto Ti surfaces have been shown to be a good strategy for controlling non-specific protein and microorganism adsorption, osseointegration and tissue-healing phenomena. For this, self-assembled monolayers (SAMs) provide a simple and precise approach to coherently modify pre-activated Ti surfaces. SAMs act as stable and well-organized intermediate structures that allow the control of the chemical functionality at the interface even on complex geometries. This is of great importance not only for controlling the interaction between surfaces, proteins, bacteria and cells, but also for serving as a stable engineering platform to additional coatings with advanced and specific properties.

For all the aforementioned, this review aims to describe the most important and recent developments on some specific approaches to create bioactive coatings on the surfaces of Ti. Indeed, this review is centered in a general surface modification strategy that includes from the typical pre-activation procedures by hydroxylation to those implied in the subsequent surface coupling of, firstly, stable intermediate SAMs and, finally, active layers. Specifically, herein, active layers formed for the chemical immobilization of active agents or for the controlled release of active compounds from polymeric platforms (such as hydrogels and multilayers) are described. Accordingly, this work summarizes the recent attempts in both strategies to achieve an active response on Ti surfaces for biomedical applications.

## 2. Pre-Activation of Ti Surface

**Figure 1:**
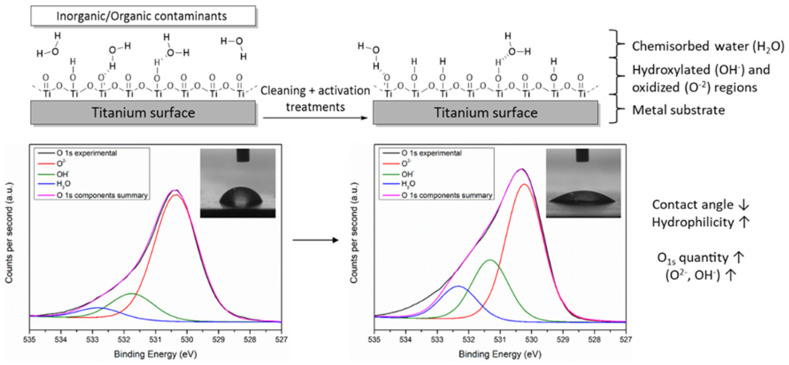
General schematic representation of the layers and chemical components of Ti surfaces before and after cleaning and activation treatments. Contact angle and XPS spectrum of O_1s_ photopeak narrow-scan with O^2−^, OH^−^, and H_2_O components are also shown as common characterizations.

Effective long-term active coating on Ti-based biomaterials requires carefully controlled surface modification from initial functionalization pretreatments [23]. Cleaning of the surface of contaminants or pollutants, and the subsequent surface activation are essential pretreatments of the surface of pristine Ti derived biomaterials to obtain active implants [20]. Under normal atmospheric conditions, a 1 to 5 nm thick passive surface oxide film is spontaneously formed in the surface of pristine Ti for corrosion protection [24]. Although this oxidized surface film is almost entirely formed of TiO_2_ (O^2−^), small amounts of active hydroxides or hydroxyl groups (OH^−^) and water are also found. Thus, the surface activation process entails specific oxidation and hydroxylation increase in the outer part of the Ti surface [25]. In this way, while the thickness of the oxidized part (TiO_2_) is maintained or even increased, numerous amounts of reactive -OH groups are created. These changes in Ti surfaces can be easily observed by contact angle measurements and XPS spectroscopy according to Lee et al. [26] and McCafferty et al. [27]. In fact, surface pre-activation promotes both a significant increase of surface hydrophilicity, and changes in Ti surface chemical composition increasing oxygen (O_1s_ ↑) and their components (O^2−^, OH^−^, and H_2_O ↑) and decreasing carbon (C_1s_ ↓) atomic percentages (Figure 1).

In addition, it is well-known that surface activation entails the improvement of many biological features of Ti-based biomaterials from biocorrosion resistance to biocompatibility [28]. In fact, the oxide layer (mainly TiO_2_) is the responsible of avoiding, on the one hand, atmospheric corrosion and, on the other hand, the release of harmful ions from implants into the human body that can result in adverse biological reactions [29,30]. Additionally, in recent years, novel investigations have shown that the mere fact of activating Ti surface could also enhance some desirable bioactive properties for potential cardiovascular, dental, and orthopedic applications: osseointegration and tissue regeneration, among others [31,32,33,34].

Nevertheless, implantation procedures and post-implantation periods involve many complex biochemical processes that could make insufficient the previously mentioned bioactive properties of oxidized Ti surfaces [6]. For this reason, with the aim of improving biomaterials performance, novel pathways to provide other beneficial properties, such as antibacterial [35], anti-inflammatory [36], drug delivery [37], self-healing [38] and tissue or wound healing [39] have been investigated for years. It is at this point in which reactive -OH groups of hydroxylated region acquire a crucial role because they provide reactivity to Ti surfaces for further chemical functionalization with active bio(macro)molecules, drugs, or other agents [40,41]. Therefore, surface activation processes apart from forming the beneficial oxide layer on Ti surfaces (TiO_2_), allow us to obtain high hydroxylation (Ti-OH) degrees that enable a higher number of functionalization points with the active agents.

**Figure 2:**
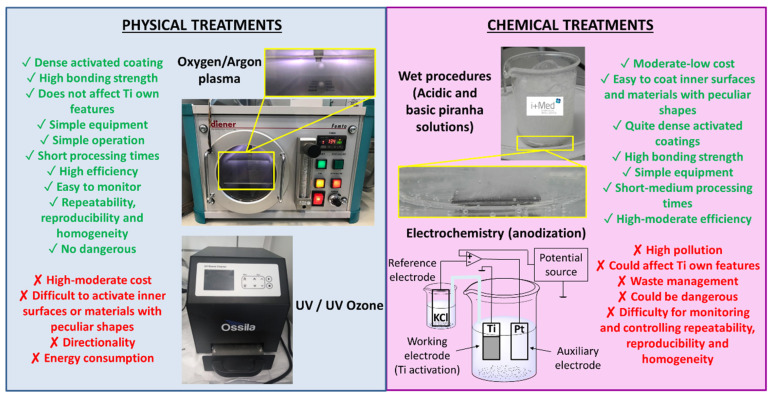
Different physical and chemical treatments for activation of Ti surface with their advantages (green) and disadvantages (red).

Accordingly, the most widely used treatments to clean and activate Ti surfaces are compared below with a special focus on the effectiveness of creating superficial active -OH groups, as a first step for the subsequent surface functionalization. In this regard, the treatments for the activation of Ti surfaces are commonly classified in physical and chemical methods as seen in Figure 2 [42]. While among physical treatments the most common methods are plasma [43] and UV/UV ozone [44], electrochemistry (anodization) [45] and wet procedures can be found among the most used chemical processes [46].

Oxygen/argon plasma is not able to activate Ti implants with complex shapes due to geometrical limitations. Despite this drawback, which restricts real-life applications, this methodology is the most employed physical approach since it is simple, reproducible, fast and environmentally friendly [47]. In particular, this type of plasma system works by increasing the hydrophilic nature of Ti surfaces by the bombardment of free radicals generated with high frequency voltages in oxygen plasma (OH^−^, O^2−^), resulting in TiO_2_ oxide layer with external Ti-OH groups [48]. Analogously, with similar advantages and disadvantages, UV or UV/ozone methods also promote hydrophilicity increase. This is carried out by two main mechanisms. One is the decomposition of hydrocarbons on the TiO_2_ coating, and the other is the conversion of Ti^4+^ to Ti^3+^, favoring Ti-OH formation owing to dissociative water adsorption [49,50,51].

Regarding chemical methods, although wet treatments are considered quite dangerous and highly pollutant, they are already the most used in industry due to their versatility to activate all type of Ti implants shapes, and their ease, simplicity and effectiveness. Among them, a wide range of different treatments can be found, such as acidic (HCl/H_2_O_2_, H_2_SO_4_/H_2_O_2_, H_2_SO_4_/HCl, …) or alkaline (NH_4_OH/H_2_O_2_) piranha solutions, and treatment with acid (HCl, H_2_SO_4_, …) or basic (NaOH, KOH, …) solutions. However, in-depth studies demonstrated that the greater quantity of -OH groups per nm^2^ was achieved using acid/H_2_O_2_ and base/H_2_O_2_ combinations rather than acid/acid combinations and only acid or basic solutions [52,53]. On the other hand, it is worth highlighting anodization, which is an electrochemical method that generates a Ti surface oxide layer via electric current under the appropriate electrolyte (KOH, NaOH, H_2_SO_4_, H_3_PO_4_, HF, …) presence [54]. In this treatment, the Ti sample acts as anode (positive working electrode) of the electrolytic cell and the auxiliary electrode (cathode) usually is composed of platinum (Pt) [55].

**Figure 3:**
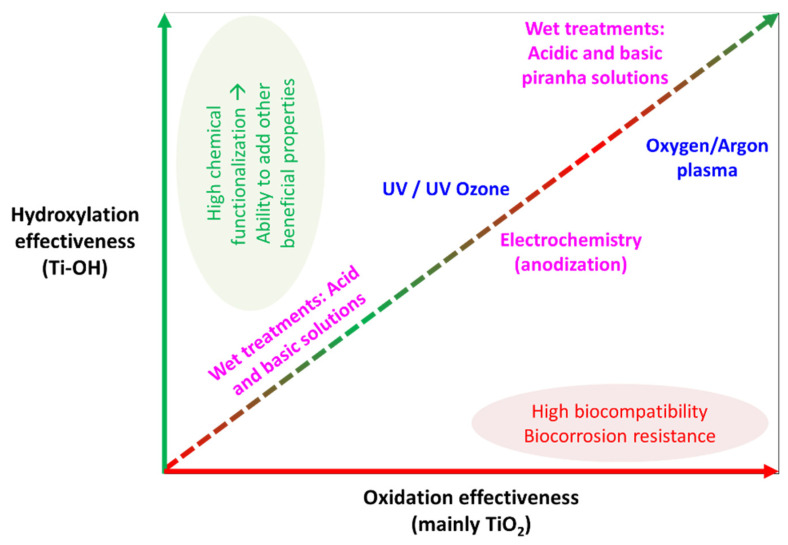
Graphic comparison of the surface hydroxylation and oxidation (TiOH/oxide ratio) effectiveness for each Ti surface activation method.

Regarding oxidation and hydroxylation effectiveness, numerous studies have compared the above-mentioned methods for an effective Ti surface cleaning and activation. One of the first studies performed almost 30 years ago on Ti6Al4V concluded that piranha wet treatments produced the highest quantity of Ti-OH groups with an intermediate level of durability, while electrochemical anodization and plasma treatments provided the best oxidation durability results with less -OH functional groups creation [56]. Four years later, McCafferty et al. quantified Ti surface activation after argon plasma treatment in 10 –OH [57] and 11 –OH groups per nm^2^ with 3.2 nm and 0.95 nm thicknesses of oxide and hydroxyl regions, respectively [27]. Recently, Son et al. [58] realized an experiment in order to compare the effectivity of UV and oxygen plasma physical treatments to activate commercially pure Ti substrates. Although both physical treatments were able to exhibit superhydrophilicity (∼10°) during more than 90 days, oxygen plasma treatment was capable to supply more quantity of TiO_2_ and Ti-OH species. Moreover, Gadois et al. [59] compared two chemical treatments for the activation of T35 Ti foil, and electrochemical anodization (H_3_PO_4_/HF electrolyte) created remarkably more hydroxyl groups fraction (15.0%) than 5 M NaOH wet treatment (7.0%). Nonetheless, in order to improve electrochemical anodization provided hydroxylation capacity, Ti surfaces are usually treated with piranha wet treatments after anodizing, thereby achieving an increase in the number of reactive -OH groups [60]. Additionally, Li et al. [61] compared UV physical treatment and electrochemical anodization (NaF electrolyte) effectiveness in commercially pure Ti plates, evidencing a higher -OH moieties introduction after UV exposure, while anodization favored TiO_2_ formation. Several studies have corroborated, after comparing oxygen plasma physical method and piranha wet treatments [62,63,64], the higher reactivity of piranha treated Ti surfaces due to the greater amount of active –OH groups in the metal oxide film, resulting in higher OH^−^/O^2−^ ratios, despite achieving a higher degree of oxidation (TiO_2_ ↑) with oxygen plasma treatments. Figure 3 briefly summarizes the effectiveness of each Ti surface activation method to create TiO_2_ or/and Ti-OH species (Ti-OH/TiO_2_ ratio).

## 3. Self-Assembled Monolayers (SAMs)

As mentioned above, the pre-activation layer is a necessary step to obtain a more easily adjustable surface. Once the hydroxyl layer is generated onto Ti surfaces, other strategies such as chemical modifications are carried out in order to obtain extensive options for further functionalization. Among chemical modifications, self-assembled monolayers (SAMs) have been frequently employed due to their high versatility [65,66]. Self-assembled monolayers are generated through the ordered assembly of adsorbed molecular components on the surface of many material and, as a result, a spontaneous thin layer is created [67,68,69]. Adsorbates include alkene/alkyne, amines, alkyl iodides, carboxylates, silanes, phosphonates and cathecol derivatives among others. The properties of the solid surfaces, including their physical, chemical, electric, bioactivity and optical properties can be modulated by incorporating the specific terminal end group of the SAMs [70]. Consequently, this type of surfaces has shown wide applicability in very different fields such as molecular sensors [71], electrochemical sensing [72,73] biosensors [74,75], preventive anti-biofouling surfaces [76], organic electronics [77,78] and tribological applications [79], among others.

Self-assembled monolayers can be classified into six groups depending on the main coupling chemistry group in the attachment: carboxylates, alkenes/alkynes, amines, silanes, phosphonates and catechols. In this review, the last three approaches will mainly be described owing to their widely use in Ti surfaces [80].

### 3.1. Silanes

Silanization has shown to be an effective and economical strategy to form bioactive coatings [81]. Organosilanes (RSiX3, R2SiX2 and R3SiX, where R is an alkyl group and X a good leaving group such as halide, alkoxy or hydride) react with surfaces rich in hydroxyl groups obtaining controlled oxide monolayers. Hydroxyl groups link steadily to silicon atoms through a covalent bond that stabilizes the monolayer. One of the main advantages of this strategy is that many commercially available silane-coupling agents allow further chemical modifications without compromising the integrity of the monolayer [80]. The introduction of reactive groups such as amino or carboxyl moieties permits the grafting of biological active molecules.

In general, regarding the structure of the silane-coupling agent three components can be distinguished: (1) the head agent that is anchored to the surface; (2) the spacer which has the ability to create the monolayer; and, finally, (3) the functional end group which tailors surface properties [67,82]. Several studies have shown that the length of the alkyl chain is crucial for the organization and stability of the SAM. In fact, the stability of the monolayer is determined by the Van der Waals interactions established between adjacent molecules [83]. As is commented above, the functional end group of the monolayer enables tailoring of the surface properties, such as chemical reactivity, conductivity, wettability, friction, adhesion, and so forth [82]. This group is, therefore, the most important part to carry out further surface reactions on alkyl-silane-based monolayers.

Two different pathways can be followed to introduce a functional end group on Ti surfaces: the direct linkage to the metallic surface of pre-functionalized organosilane, or the chemical modification after SAM is formed. This second strategy has been revealed to be a better route due to several reasons. Firstly, the order of the monolayer is not affected by additional surface modifications as long as the immobilized SAM is stable enough [82] and, consequently, also the post-functionalization prompts. Secondly, different chemical reactions can be employed to modify the same surface, which offers more options for introducing various functional groups. In addition, this methodology is extremely simple and there is no need to synthesize and purify new tailor-made precursors since the most employed molecular precursors that form well-defined monolayers are easily accessible [81].

**Figure 4:**
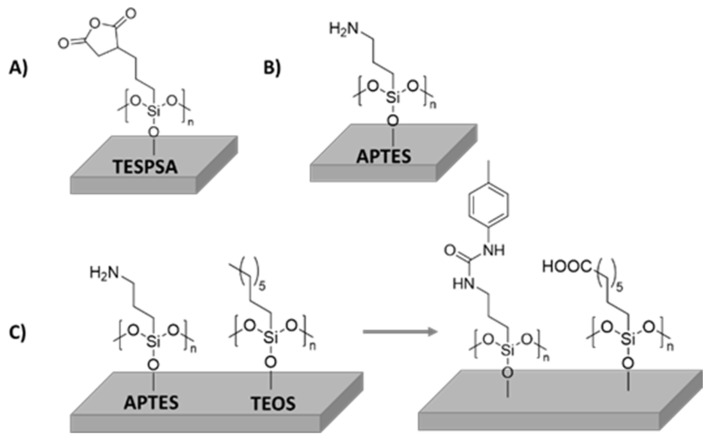
Schematic representation of silanes covalently immobilized onto Ti surfaces: (**A**) TESPSA immobilization onto Ti dental implant, (**B**) APTES immobilization onto Ti6Al4V alloy and (**C**) APTES and TEOS immobilization onto Ti6Al4V and their further functionalization.

Despite silane-type coatings being mainly used to bind biological active compounds to Ti in order to obtain bioactive surfaces, some silane coatings have demonstrated antibacterial properties or improved cell adhesion (Figure 4A). An example of this was reported by Godoy-Gallardo et al. In this work, the immobilization of triethoxysilylpropyl succinic anhydride (TESPSA) onto Ti dental implants with antimicrobial properties was described. For this purpose, Ti substrates were firstly activated with NaOH solution in order to create hydroxylated surfaces that were subsequently functionalized with TESPSA silane. The successful conjugation of TESPSA was analyzed by means of scanning electron microscopy (SEM), where a sodium titanate surface was observed, and an increase of silicon signal (Si-O 8.4% for Ti-N-TESPSA) was confirmed by X-ray photoelectron spectroscopy (XPS) after silane immobilization. Furthermore, a lactate dehydrogenase assay concluded that TESPSA did not have a negative effect on the viability of human fibroblasts. Importantly, the in vitro effect of modified surfaces against *Streptococcus sanguinis*, *Lactobacillus salivarius* and oral plaque were studied using a viable bacterial adhesion assay. A significant reduction was obtained in all cases but, as expected, there was a difference in effectiveness between samples against simple mono-species biofilm (ratio dead/live of 0.4) and complete oral biofilm (ratio dead/live of 0.6). Nevertheless, this approach still has a great potential to provide antibacterial properties to dental implant [84,85].

Another example of silane activity was reported by Abraham Rodriguez and coworkers. They presented a reproducible methodology to obtain a cross-linked polymer-type brush structure of covalently-bonded aminoalkylsilane chains on Ti6Al4V (Figure 4B). They functionalized Ti6Al4V alloy with 3-aminopropyltrimethoxysilane (APTMS) achieving a high density coated surface, which could be resilanized. They studied *Staphylococcus* adhesion and biofilm formation compared to the pristine Ti6Al4V oxidized surface. Biological tests showed that the modified Ti-alloy is appropriate for biomedical implants and prostheses. According to human primary osteoblast behavior, aminosilanized samples displayed a similar cytocompatibility to that of the alloy. Moreover, bacterial assays with *S. epidermidis* cultures indicated that aminosilane layers exhibited protection against adhesion [86].

Another example of silanized Ti coating was described by Hasan et al. [87]. In this work, a study of the effect of Ti6Al4V surface functionalization on protein adsorption and cell behavior was reported. They prepared five different SAM surfaces (amine, octyl, mixed [1:1] ratio of amine:octyl], hybrid, and COOH). TiO_2_ samples were firstly activated with piranha treatment to obtain hydroxylated surfaces, then amine silane (APTES) and octyl silane (TEOS) and mixtures of them were employed as coupling agents (Figure 4C). Hybrid SAMs were synthesized by immersing NH_2_-modificed surfaces into p-tolyl isocyanate solution, and carboxylic acid SAM was obtained by oxidizing the CH_3_-modified surface. All samples were characterized by Fourier transform infrared-attenuated total reflection (FTIR-ATR) spectroscopy, contact angle goniometry, profilometry, and field emission scanning electron microscopy (FESEM), corroborating the performed modifications. Bovine serum albumin (BSA) and fibronectin (FN) were employed in order to determine protein adsorption and cell adhesion, respectively. The authors found that the amount of adsorbed BSA was higher in more hydrophobic surfaces, obtaining a maximum absorption value on the octyl surface. However, the adsorbed amount of FN was found to increase in more hydrophilic surfaces, achieving the maximum adsorbed mass on COOH surface. Due to maximum cell adhesion and proliferation, larger nuclei area and less cell circularity, hybrid samples showed to be the most promising surfaces compared to unmodified T6Al4V to be potentially employed for tissue engineering applications.

### 3.2. Phosphonates

The employment of phosphates and phosphonates is another cost-effective strategy to link biomolecules to metal surfaces such as TiO_2_ or Ti alloys in order to achieve bioactive surfaces. As silanes, phosphonates have shown biological compatibility on their own, without needing to incorporate active agents. In fact, phosphonate linkers are similar to silane coupling agents, although they present the significant advantage of being more stable in aqueous environments at physiological pHs due to their hydrolytic stability. The use of robust and stable coatings under physiological conditions in biomedical applications is of high interest, and thus, phosphates or phosphonates have aroused a great interest to develop stable SAMs onto metal oxides [80].

**Figure 5:**
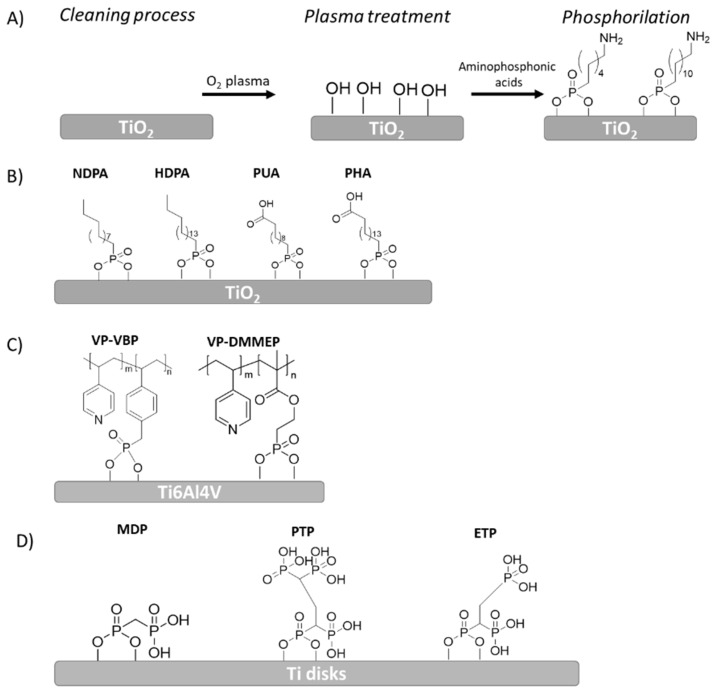
Schematic representation of different alkyl phosphonic acid immobilization onto Ti or its alloys: (**A**) functionalization strategy of aminophosphonates with different leghts onto Ti implant, (**B**) NDPA, HDPA, PUA and PHA immobilization scheme onto TiO_2_ implant, (**C**) Different phosphonate copolymers immobilization onto Ti6Al4V, (**D**) MDP, PTP and ETP phosphonic acids immobilization onto Ti disks.

Paolo Canepa et al. employed an aminophosphonate for a first step functionalization of TiO_2_ layer on Ti. This phosphonate molecule has a group on one end, which can be exploited for coupling with the oxide surface, and an amino moiety on the other end which enables further functionalization of the surface. By combining different surface-sensitive experimental techniques, they found a discontinuous monolayer where the molecules were covalently coupled to the TiO_2_ surface. These authors studied the deposition of aminophosphonates with different chain lengths (6 and 12 methylenes) (Figure 5A) and also found that larger chain molecules provided a more ordered layer, especially after thermal annealing. In order to preserve unreacted amino groups to be used in further functionalization, it was essential to perform the annealing process in an inert atmosphere. Indeed, their preliminary studies indicated that unreacted amino groups could be anchored to single amino acids by using aminododecilphosphonic acid [88].

Similarly, Lan et al. [89] developed phosphonate self-assembled monolayers for biomedical applications. The purpose of the study was to fabricate biocompatible SAMs on a TiO_2_ layer using four kinds of phosphonic acid solutions via self-assembly technology and investigate their effect on surface properties and biomechanical behavior of the modified surfaces. After the activation of TiO_2_ surfaces with plasma treatment, the samples were separately submerged into four kinds of phosphonic acids: hexadecylphosphonic acid (HDPA), decylphosphonic acid (NDPA), 11-phosphonoundecanoic acid (PUA) and 16-phosphohexadecanoic acid (PHA). These phosphonic acids present different chain lengths and terminal functional groups (Figure 5B): (i) CH_3_ as a hydrophobic and non-reactive group (HDPA-SAM/TiO and NDPA-SAM/TiO samples) and (ii) COOH as a hydrophilic and reactive group (PUA-SAM/TiO and PHA-SAM/TiO samples). All samples were analyzed by means of SEM, XPS, contact angle and contact stiffness. Based on the XPS results, after being coated with different SAMs, the binding energy of O 1s indicated that the component characteristics of P=O (≈532 eV), P–Ti–O (≈530 eV), and O–Ti (≈529 eV) were bonded in the surface layer. It is well known that the orientation of adsorbed molecules onto surface and the density of the surface layer determine the hydrophobic-hydrophilic balance of a surface. For this reason, according to contact angle results, as expected, surfaces with CH_3_-terminated SAMs were slightly hydrophobic, they were even shown to be more hydrophobic than the TiO_2_ surface due to the formed homogeneous and compact SAM. On the other hand, surfaces with COOH-terminated SAMs were more hydrophilic compared to the TiO_2_ surface. In the case of stable, compact SAMs where alkyl chains are connected via Van der Waals interactions, only a terminal functional group is in direct contact with the solvent drop. On the other hand, the SAM-coated surfaces exhibited a decrease in stiffness as a consequence of the formation of the bisphosphonate monolayer, which could disperse stress concentration, hence decreasing the stress shielding effect. Thus, the formation of stress shielding can cause a loss of bone tissue around the implant and, consequently, poor osseointegration. This effect typically occurs when metal implants are used to repair or joint bones as a consequence of the higher stiffness of the implant, leading to bone loss due to restricted physiologic loading of the bone.

Noah Metoki and coworkers presented a completely different strategy to anchor phosphonic acids onto the Ti implant. They analyzed both active (electro-assisted) and passive (adsorption) approaches for the modification of Ti6Al4V using alkylphosphonic acid such as hexylphosphonic acid (HPA), NDPA, tetradecylphosphonic acid (TDPA) and HDPA. They demonstrated that electrochemically assisted monolayers, besides being assembled faster, showed better control over surface properties such as a superior degree of order and a higher packing density. The electrochemically absorbed SAMs also displayed better blockage of electron transfer across the interface and, consequently, better corrosion resistance. Alkylphosphonic acid self-assembled monolayers with different chain lengths were assembled on Ti6Al4V via either chemisorption or electrochemical deposition. It was found that long-chain acids formed closer packed monolayers, resulting in more hydrophobic surfaces. More significant was the reduction of electron transfer across the interface, thus, influencing the corrosive behavior [90].

Other examples of the use of phosphonate, such as 4-vinylpyridine (VP) with vinylbenzylphosphonate (VBP) or dimethyl(2-methacryloyloxy-ethyl) phosphonate (DMMEP) were described by Calliess et al. (Figure 5C) [91]. They synthesized a variety of copolymers with different ratios of VP and phosphonate monomers through free radical polymerization onto Ti6Al4V samples. These copolymers were further functionalized with 1-bromohexane to form *N*-hexylpyridinium bromide groups (HexVP). The contact angles of the copolymer coatings varied in the range of 56–74° depending on the composition of polymers, so, in this context, it was showed that a higher content of polar HexVP groups provided a less hydrophobic surface. On the other hand, an increase of DMMEP exhibited more hydrophilic values, thus, the homopolymers of DMMEP showed better non-fouling properties as well as good antibacterial activity. For this reason, an increase of DMMEP in the copolymers with a higher content of DMMEP showed a reduction of adherent bacteria up to 95% compared with blank Ti controls, and antimicrobial activity against *S. epidermidis* and *S. aureus*. However, a decrease in biocompatibility was found in the same copolymer, although no cytotoxic effect could be observed in the period of cultivation.

In the same way, Pfaffenroth and coworkers synthesized different copolymers of 4-vinyl-N-hexylpyridinium bromide (HBVP) and dimethyl(2-methacryloyloxyethyl) (DMMEP) phosphonate self-assembled to form ultrathin layers on Ti surfaces (Figure 5D). They demonstrated that phosphonate-coated Ti samples had antimicrobial activity and good biocompatibility. The antimicrobial effect of the surface was enhanced by an increase in the content of DMMEP within the copolymer, same as Calliess et al. demonstrated above. Meanwhile, the introduction of hydrophilic monomers improved the antibacterial effect of the copolymers compared to poly(HBVP) homopolymer and, in particular, compositions with low amounts of HBVP showed strong effects [92].

Viornery et al. [93] synthesized several phosphonic acid that were grafted onto Ti disks in order to increase the chemical interaction between the implant and bone tissue. Three phosphonic acids were grafted: methylenediphosphonic acid (MDP), propane-1,1,3,3-tetraphosphonic acid (PTP), and ethane-1,1,2-triphosphonic acid (ETP) (Figure 5D). The bioactivity of the modified Ti disks was evaluated by incubating these disks in a physiological solution (Hank’s balanced salt solution (HBSS)) for 1, 7 and 14 days. Modified surfaces showed only slightly higher calcium levels in the XPS analysis compared to the reference Ti-P surface. Among them, the surface modified with ETP (Ti-P + ETP) induced the highest calcium phosphate deposition after 14 days incubation.

Furthermore, Petrovic et al. [94] performed theorical simulations of the formation mechanism of the aleondronate sodium coating on Ti implant surface. According to quantum chemical calculations, the interaction between the aleondronate and Ti surface was carried out more spontaneously by exergonic process when aleondronate molecules bind directly to Ti surface through two strong bonds via amine (-NH_2_) and phosphonate (-PO_3_H) group. This stable structure included extra hydrogen bonding, which provided good coating stability in artificial saliva media for 7 days. These interactions were also corroborated with contact angle and XPS measurements. Additionally, some –NH_2_, –COH and –PO_3_H groups remained free in the formation of the coating, which lead to a more hydrophilic implant surface.

### 3.3. Catechols

Catechol coatings have gained much attention in recent years due to the simplicity and efficiency with which they can anchor biomolecules onto a substrate. Similar to silanes and phosphonates, catechols and their derived compounds interact successfully with almost any kind of surface. In fact, they can be easily self-assembled on various inorganic and organic devices, metals, ceramics and even polymers, despite having a very simple structure as it only presents a benzene ring with two-hydroxyl moieties [95]. Their mode of attachment is inspired by the adhesive proteins secreted by mussels. In this regard, mussels can resist erosion of the sea owing to the firm adhesion to solid surfaces [96]. It has been observed that mussel’s amino acids, which are rich in 3,4-dihydroxy-l-phenylalanine (DOPA) and lysine amino acids are the main reason for their binding strength, since DOPA forms strong covalent and noncovalent interactions with the substrate [95,97]. This strategy opens a new route to modifying various substrates and preparing functional composite materials by simple chemistry [98]. The good adhesion produced by catecholic compounds has originated great interest in exploiting this type of compound in order to enhance interfacial adhesion of synthetic materials. Hence, catecholic derivatives provide another novel and useful alternative for surface immobilization. Despite the fact that polydopamine coating provide itself antimicrobial activity, it is often complexed with antimicrobial agents to improve biological response. For this reason, examples of the use of this type of SAM are described in more detail in the following section.

## 4. Active Layer

SAMs have been shown in several examples to be able to promote osseointegration, reduce oxidative character or even improve antimicrobial activity in a passive way, that is, by altering the physicochemical properties of the substrate and, accordingly, the interactions with cells and bacteria. However, the effectiveness of passive coatings is limited, especially reducing bacterial adhesion [99], which has endorsed the interest in active alternatives. Active approaches are based on the incorporation of biological active compounds on substrate surfaces for achieving improved biocompatibility and better protection against bacteria or other microorganisms. Such active coatings can be designed according to two different strategies: (i) immobilization of active molecules by chemical linkage, and (ii) physical loading, and subsequent release of active agents on polymeric platforms.

### 4.1. Immobilization

The immobilization of several drugs and biological active complexes can be carried out following different chemical reactions such as nucleophilic substitution, click chemistry, photochemical reactions and others. The first two types of reaction are considered the most relevant and most broadly employed in the last decade.

#### 4.1.1. Nucleophilic Substitution Reactions

Nucleophilic substitution reactions have been widely used to achieve chemically active surfaces. In general, an electron-rich nucleophile such as H_2_O, NH_3_, –OH, –N_3_, –CN, etc. attacks to an electrophile, where the suitable leaving group of the electrophile is exchanged by the nucleophile. Nucleophilic substitution reactions can either be used to bind a self-assembled monolayer onto a substrate or can be employed as a modification reaction of a self-assembled monolayer that provides a suitable functional group [100,101].

One frequently used nucleophilic substitution to functionalize SAMs is the *N*-hydroxysuccinimide (NHS) reaction, which is very popular due to its versatile applicability. The NHS ester is probably the most commonly used functional group in activation chemistry for generating reactive acylating agents. The formed reagents of NHS or NHS ester-containing molecules can further react with nucleophiles forming an acylated product and release the NHS or NHS-containing byproducts. This strategy is commonly applied to the following two approaches: (i) using NHS or NHS derivatives to react with carboxylate functionalized surfaces or (ii) direct attachment of the NHS derivatives onto the surfaces. Both modified surfaces can further react with sterically accessible amine-terminated reactants generating an amide bond.

**Figure 6:**
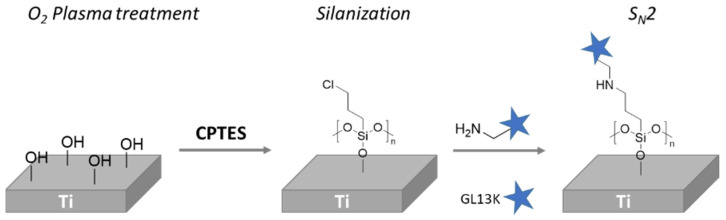
Schematic representation of GLK13K protein immobilization onto Ti surface.

Holmberg et al. developed a novel Ti coating made by the immobilization of antimicrobial peptide (GL13K), which has applications for preventing infection-related implant failures in dentistry and orthopedics. Ti samples were pre-activated with O_2_ plasma treatment and followed by silanization with 3-(chloropropyl)-triethoxysilane) (CPTES) (Figure 6). The conjugation of GL13K was carried out under argon conditions via S_N_2 reaction. As a result, a highly hydrophobic and strongly anchored GL13K coating was achieved, which presented resistance to mechanical, thermochemical and enzymatic degradation. Additionally, the GL13K coatings exhibited a bactericidal effect and, thus, the number of viable bacteria was significantly reduced compared to control surfaces. The cytocompatibility of the surface was determined with gingival fibroblast and an adequate proliferation of osteoblasts was observed [102].

**Figure 7:**
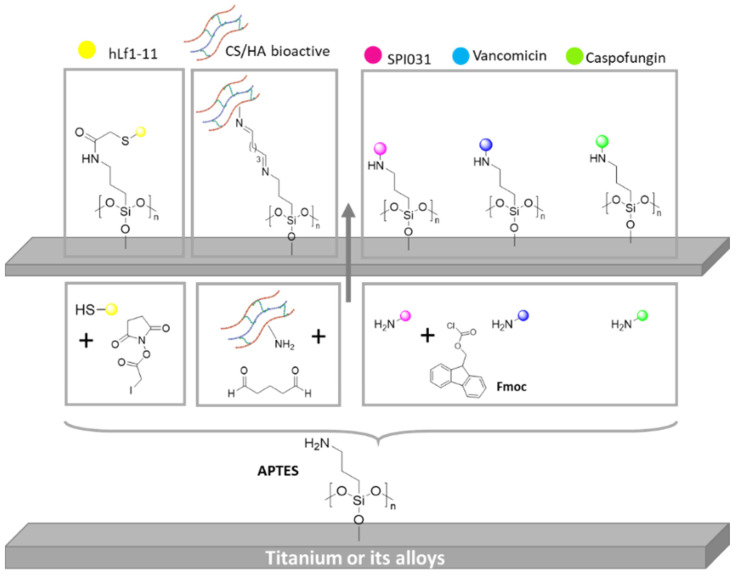
Schematic representation of different bioactive compound immobilization through silanization.

A similar example of this approach was described by Godoy-Gallardo et al. [103]. Smooth Ti samples were coated with hLf1−11 peptide, an antibacterial peptide, under two different conditions: (I) a silanization with 3-aminopropyltriethoxysilane (APTES) and (II) surface functionalization via atom transfer radical polymerization (ATRP). For this purpose, Ti samples were previously activated with oxygen plasma. Then, the amino group of the silane was modified with iodoacetic acid *N*-hydroxysuccinimide ester through acetylation reaction. After that, hLf1-11 peptide was anchored by S_N_2 reaction, in which the thiol end group of hLf1-11 reacted with iodine atom of silane (Figure 7). The authors found that samples modified with ATRP methods showed a higher decrease in bacterial attachment compared to silanization. This effect is likely due to the capacity to immobilize more peptide on the surfaces using polymer brushes and the nonfouling nature of a polymer PDMA segment.

Similarly, Chen et al. [104] employed 3-aminopropyltriethoxysilane (APTES) anchor to graft melimine, a synthetic antimicrobial peptide, onto Ti surfaces. In this study, Ti surfaces were first amine-functionalized with APTES and followed by a bifunctional linker 4-(*N*-maleimidomethyl) cyclohexane-1-carboxylic 3-sulfo-*N*-hydroxysuccinimide ester(Sulfo-SMCC) to obtain a maleimide functionalized surface. Melimine was anchored to the surface via thio-ether linkage employing a Michael addition reaction of the cysteine at its N-end groups with maleimide moiety. The in vitro antimicrobial activity of melimine-coated Ti surfaces demonstrated that melimine coating reduced significantly bacterial adhesion and the biofilm formation of *P. aeruginosa* (up to 62%) and *S. aureus* (up to 84%) compared to Ti substrates.

Viena Vyas and coworkers employed a different strategy based on a glutaraldehyde (GLU) reaction to anchor chitosan (CS) and hydroxyapatite (HA) onto commercially pure Ti surfaces (cpTi). They grafted these bioactive molecules in order to promote osteoblast adhesion and bone growth. For this purpose, they pre-activated cpTi surfaces by piranha treatment and, then, samples were silanizated with APTES and formylated with GLU (Figure 7). Finally, CS/HA biocomposite was reacted to modify cpTi surfaces via nucleophilic addition. The coated cpTi samples exhibited a higher hydrophilicity along with a significant enhancement in bioactivity. Moreover, they showed improved hemocompatibility as well as better osteoblast cell adhesion and proliferation [105].

Gerits et al. [106] presented Ti substrates modified with an antibacterial agent named SPI031, covalently linked (SPI031-Ti) via silane anchor. They demonstrated that SPI031-Ti coated samples exhibited in vitro less bacterial adhesion compared to control-Ti disks. They showed that coated samples were less active against the Gram-negative bacterium aeruginosa than against the Gram positive bacterium aureus. The same team grafted vancomycin (VAN) and caspofungin (CAS) onto Ti substrates using, again, a silane anchor strategy to immobilize, separately, the drugs onto Ti surfaces (Figure 7) [106]. In both cases, an exceptional antibacterial activity was observed. VAN functionalized samples exhibited a significant in vitro reduction of biofilm formation when S. aureus was employed. On the other hand, a complete prevention of biofilm formation was analyzed when CAS-Ti samples were employed.

Regarding phosphonate based SAMs, Jörg Auernheimer et al. [107] modified Ti implants with a tailor-made cyclic-RGD peptide, thus allowing them to bind to specific integrin receptors on the cell surface through multimeric phosphonates. For this purpose, they synthesized cyclic pentapeptide with four phosphonic acid end groups in order to anchor to the Ti surface.

Julien Amalric et al. [108] functionalized Ti6Al4V alloy with a self-assembled phosphonate monolayer that then reacted with silver thiolate species in order to prevent bacterial adhesion. Ti and stainless-steel substrates were firstly modified by grafting with mercaptododecylphosphonic acid (MDPA) followed by reaction with silver nitrate_._ Despite surfaces containing a very low silver quantity, MDPA + AgNO_3_ monolayers strongly decreased the bacterial adhesion of the surface compared to the non-coated Ti or stainless-steel substrates or to the samples modified by MDPA only: a 3- to 5-log reduction in the number of viable adherent bacteria was found for the four bacterial strains tested (*E. coli*, *S. aureus*, *S. epidermidis* and *P. aeruginosa*). The efficiency of MDPA + AgNO_3_ monolayers confirmed the importance of the localization of the bactericidal species directly at the surface.

Catechol based SAMs have also been employed in nucleophilic substitution reactions, Xuefeng Hu et al. functionalized Ti surface with vascular endothelial growth factor (VEGF) employing catechol-anchoring strategy. They modified either carboxymethyl chitosan (CMCS) or hyaluronic acid (HAC) with dopamine in order to graft them covalently onto Ti surface. According to antibacterial assays against *S. aureus*, a Gram positive bacteria, both derivatized polysaccharide substrates showed a significant decrease in bacterial adhesion. Researchers concluded that carboxymethyl chitosan provided a higher antibacterial efficacy than hyaluronic acid. Indeed, the number of viable *S. aureus* cells on grafted CMCS and HAC decreased to 16% and 54% respectively compared to control Ti. Moreover, the immobilization of VEGF in both strategies resulted in an enhancement of osteoblast and endothelial cell functions [109].

Another example is described by Andrade and coworkers, who prepared different polymeric antibacterial coatings employing catechol-based strategy. They developed poly(*N*-vinyl pyrrolidone) (PVP), hyaluronic acid (HA) and chitosan (CHI) Ti6Al4V surface coatings. The adhesion of these coatings to Ti6Al4V substrates were carried out after the conjugation of these polymers through anchor group. These surface modifications were characterized by XPS contact angle measurements and atomic force microscopy. In addition, the stability of CA-conjugated polymeric coatings was compared to the coatings formed with unconjugated polymers. Finally, the cytocompatibility against Gram-positive and Gram-negative strains on coated Ti6Al4V substrates was assessed confirming the effectiveness of these polymeric coatings against bacterial infections for future applications in protecting biomedical implants [110].

#### 4.1.2. Click Chemistry

Lately, click reactions have proven to be a very useful approach for bioconjugating molecules on different surfaces. The click chemistry term was firstly introduced by K. Sharpless and refers to a full class of reactions that are high yielding stereospecific and wide in scope. Moreover, the reaction conditions as well as the purification processes are simple, due to the use of solvents that are easy to remove or even, in some cases, the reactions are carried out in the absence of them. The reactions provide quantitative conversions and generate no-byproducts or only byproducts that can be removed without chromatography. A bunch of chemical reactions belong to this group such as the azide–alkyne cycloaddition reaction, thiol-ene additions and Diels–Alder reactions among others [111,112,113,114].

**Figure 8:**
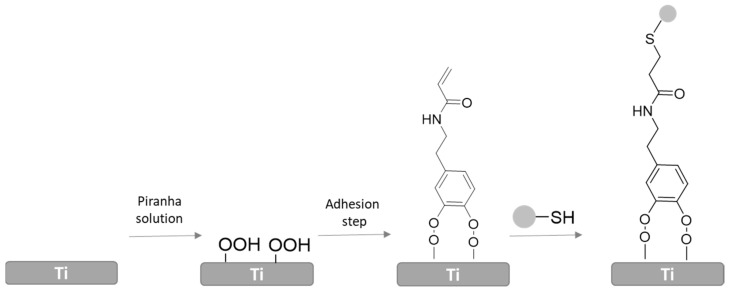
Schematic representation polyNaSS immobilization via catechol strategy and click chemistry.

Lin et al. [115] presented a method for covalent multi-biofunctionalization of Ti surface with mixtures of peptides at any desired ratios using click chemistry (Figure 8). They employed APTS silane with alkyne terminal functionalization and pre-activated peptide, demonstrating that the optimization of the type and ratio of the peptides on Ti surface resulted in an excellent antimicrobial activity as well as good biocompatibility.

A similar example was labelled by Chen et al. [116] who studied the functionalization of antimicrobial peptides (AMPs) onto Ti implants in order to prevent bacterial infection. In this study, a “clickable” Ti surface was developed by using a silane-coupling agent with an alkynyl group. The antimicrobial Ti implant was obtained through the reaction between the “clickable” surface and azido-AMPs (PEG-HHC36:N3-PEG12- KRWWKWWRR) via Cu (I)-catalyzed azide−alkyne cycloaddition (CuAAC). In vivo assay demonstrated that this implant could kill 78.8% of *S. aureus* after 7 days. Thus, this method has shown great potential for the preparation of long-term antimicrobial Ti implants and the prevention of infections in the clinic.

Andras Heijink et al. functionalized the Ti implant surface with the Arg-Gly-Asp tripeptide (RGD) in order to facilitate osteoblast attachment for improved implant fixation in the laboratory. They studied the histomorphometric and mechanical performance of Ti implants coated with RGD using self-assembled monolayers of phosphonates (RGD/SAMP) and implants coated with RGD using the more conventional thiolate-gold interface (RGD/thiolate-gold). According to the results, RGD/SAMP-coated implants showed a greater growth on bone and implant fixation than RGD/thiolate-gold-coated ones [117].

Florian Rechenmacher et al. [118] presented a versatile click chemistry-based molecular toolkit for the bio-functionalization of materials to selectively control integrin-mediated cell adhesion. For this purpose, they modified RGD peptidomimetics compounds via click chemistry that were immobilized onto Ti.

Chouirfa and coworkers developed an antimicrobial surface by immobilization of Polysodium styrene sulfonate (PolyNasS-polyanion) onto the Ti surface through dopamine strategy. For this purpose, they first activated the Ti surface with piranha solution and it was further modified with previously synthesized catechol derivative (Figure 8). After, thiolated polyNaSS polyanion was grafted to the surface by thiol-ene reaction. ToF-SIMS and XPS measurements demonstrated the efficacy of the new approach to graft bioactive polymers with well-defined molecular weight onto the Ti surface. They analyzed the in vitro biological response of different polyNaSS surfaces employing *S. aureus,* and they concluded that a bulkier polyanion provided a higher bacteriostatic effect. Moreover, these surfaces showed a positive response against *S. aureus* and authors determined a significant effect of molecular weight of the polyanion [119].

Watson et al. demonstrated that Ti surfaces can be successfully functionalized via the CuAAC reaction by utilizing a catechol-azide clickable platform. For this purpose, they prepared azide terminal dopamine anchor in order to be grafted covalently onto previously activated Ti plates. Then, derivatized Zonyl and ferrocene were immobilized by CuAAc reaction. Contact angle and electrochemical and XPS measurements concluded the efficiency of functionalizing the azide-terminated catechol layer via click chemistry. The functionalization of Ti implants with therapeutic agents using this strategy is in progress; however, in particular, this work is relevant because the followed strategy can be used as a versatile platform for grafting biomolecules onto titanium devices [120].

### 4.2. Release-Based Polymeric Coatings

Another promising strategy to create active surfaces on Ti substrates is the controlled release of active agents from a polymeric platform previously anchored [121]. Today, polymers can be processed in different morphologies to be used as release-based coatings on metallic surfaces, for example, microparticles, nanoparticles, micelles, liposomes, nanofibers, nanotubes, films, multilayers, and hydrogels [122,123]. However, the literature highlights the versatility of hydrogels and multilayers to provide smart coatings suitable to act as active agents reservoirs that, by means of a controlled release, enable the desired response in the surface [124,125].

In these release-based systems, the bioactive properties are achieved thanks to the entrapped bioactive molecules that are released in a controlled space-time manner in the therapeutic target. Drugs, proteins, peptides, growth factors, inorganic or polymeric nanoparticles, and nucleic acids among others, are recognized as widely used bioactive compounds in implantology area [126,127,128] since they are capable to modulate metabolic processes and provide biomaterials with outstanding biomedical properties.

Regarding the chemical composition of hydrogels and multilayer coatings, they have been historically composed of synthetic polymers but, in the last decade, the use of biopolymers to construct these smart structures have been encouraged, specially polysaccharides [129]. In fact, they are abundant in nature, biodegradable, biocompatible, renewable, biologically active and possess low toxicity [130]. In addition, the large number of different functional groups throughout their chemical structure, the ease to be modified and functionalized, and the facility to acquire three-dimensional complex conformations, make them suitable candidates for preparing a wide variety of release-based hydrogel or multilayer coatings [131].

There are many advantages of controlled release-based coatings over immobilization of active agents. One is the development of a more stable, not sudden, uniform and prolonged release over the time reducing harmful side effects in patients [132]. In this way, sustained release-based coatings extend the therapeutic effect of bioactive substances and diminish the over-excessed concentration peaks of conventional methods, demonstrating an improved pharmacokinetic profile [133]. Secondly, while immobilization techniques need controlled breakage and reestablishment of chemical bonds to release active agents, release-based platforms require simpler mechanisms (diffusion, swelling, degradation) that do not need to modulate specific linkages. On the contrary, immobilization methods enable high specific release approaches.

The mechanisms of these release-based systems are studied by numerous mathematical models available in literature [134,135]. The Korsmeyer–Peppas semi-empirical model [136,137] is known to be one of the most appropriate for studying the release of active agents from polymeric platforms as hydrogels and multilayer systems. In this model [138,139,140,141] the active agents release mechanisms from these matrices are generally governed by diffusion or degradation processes, or a combination of both.

Taking into account all the aforementioned, the latest developments in hydrogel- and multilayer-based polymeric coatings on Ti surfaces to provide an effective loading and sustained release of active agents for biomedical applications are summarized below.

#### 4.2.1. Hydrogel Coatings

**Figure 9:**
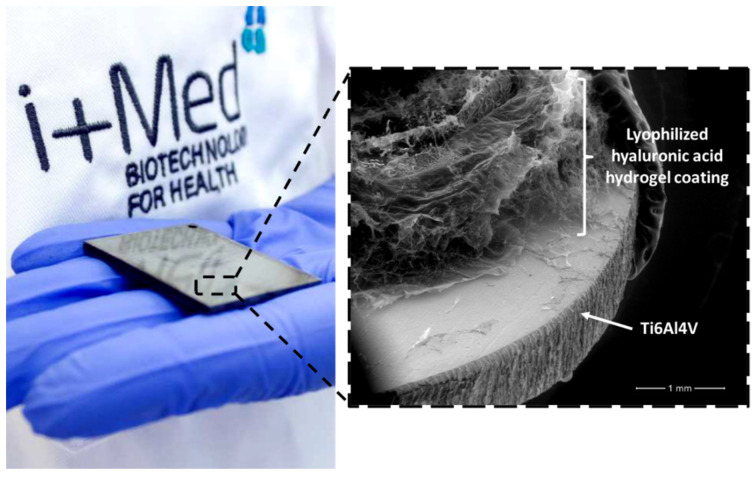
Example of a hyaluronic acid hydrogel coating on Ti6Al4V surface for potential active agents delivery.

Biopolymer-based hydrogels coatings benefit from hydrogels hydrophilic three-dimensional elastic porous microstructure to absorb large quantities of water or biological fluids loaded with active agents [142]. In that way, the amount of active agent that is loaded and steadily released through the stable hydrogel network (from hours to months) is easily controlled and can be varied from specific low- to high-doses [143]. In addition, comparing them with multilayer coatings, hydrogel coatings could possess in general greater loadings and higher ability for high molecular weight biomolecules [144]. In Figure 9, macroscopic and SEM photographs of an active coating of Ti6Al4V substrate based on hyaluronic acid hydrogel developed by i+Med S. Coop. research group can be seen.

Moreover, the higher stability of hydrogels in physiological conditions makes diffusion the predominant release mechanism of this kind of coating [145]. Nevertheless, the possibility of controlling the physicochemical features of hydrogels (swelling capacity, pore size, biodegradability, and crosslinking density) in accordance with those of active agents (stability, molecular weight, acid dissociation constant (pKa), octanol-water partition coefficient (log K_ow_) and solubility) provides the ability to modulate stability and release profiles that is of great interest within the new personalized therapies [146].

However, despite some efforts having been made to promote the anchoring of previously formed macroscopic hydrogels to Ti surfaces, even after the introduction of Ti-OH reactive groups and anchoring SAMs, the huge swelling ratio and macroscale thickness of these polymeric networks restrict their adhesion and bonding to Ti surfaces [147]. This adhesion can be measured as the resistance to unstick hydrogel coatings from Ti substrates and quantified as the energy needed to remove a unit area of hydrogel coating (J/m^2^). However, as the development of macroscale hydrogel coatings onto metallic substrates in biomedical sector is still a challenging approach, the tribological characterization of these coatings has not yet been widely reported [148].

Indeed, other strategies have been followed to achieve strong and resistant hydrogel adhesion. In situ hydrogel coating formed on Ti surfaces has shown the most promising results till date [149]. However, this method requires exhaustive purification steps since unreacted free monomers, crosslinkers and initiators could increase coatings’ toxicity. Thus, the removal of these residual molecules before their biomedical real-life application is imperative, the dialysis process being the most common procedure to remove these harmful molecules [138,150].

Some recent examples of hydrogel coatings with high adhesion to Ti surfaces developed for the controlled delivery of bioactive agents can be found in the literature. Wu et al. reported a nano-silver loaded hydrogel coating composed of 3D printed chitosan and gelatin polysaccharides with the aim of improving Ti biological fixation interface in artificial joint replacement [151]. According to the results, chitosan-gelatin coating showed high bonding strength to Ti substrate and in addition, hydrogel bonding was able to increase antibacterial activity thanks to the release of nano-silver. In this case, adhesion mechanism was based on the covalent and H bonding between chitosan/gelatin hydrogel and silane anchor monolayer previously assembled onto a Ti activated surface.

Soylu et al. [152], however, used catechol groups to bind a gentamicin-loaded agarose hydrogel on Ti6Al4V surfaces. The prepared hydrogel exhibited excellent adhesion properties apart from a potential antibacterial activity for application in spinal implants.

Sani et al. [153] employed a bioadhesive gelatin hydrogel loaded with antimicrobial peptides for the treatment of peri-implant diseases. This bioadhesive hydrogel showed significantly higher adhesion to physiological tissues and Ti surfaces than other commercial adhesives. More examples of active hydrogel coatings developed onto Ti surfaces for the controlled release of bioactive compounds are summarized in Table 1.

#### 4.2.2. Multilayer Coatings

**Figure 10:**
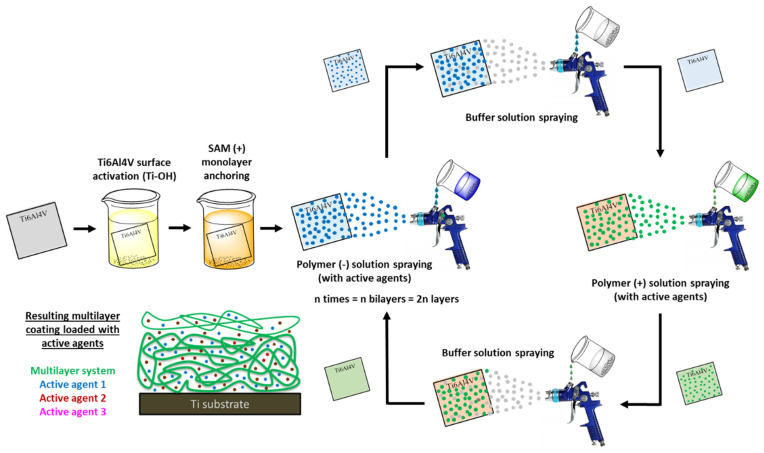
General schematic representation of LbL assembly by spray-coating method of positively- and negatively-charged polymers onto Ti6Al4V surface with loaded active agents.

Another versatile approach to developing coatings with self-controlled active agents release ability is a multilayer system built up. Indeed, Ti surfaces are covered using coating methods based on layer-by-layer (LBL) technique (e.g., dip-, spray- and spin-coating), which are fast, easy to use and adjust to any kind of substrate (Figure 10) [176]. The most commonly applied multilayer coatings in biomedical area are those based on electrostatic interactions; in fact, electrostatic attraction between oppositely-charged polymers leads to the creation of polyelectrolyte multilayers or polyelectrolyte complexes (PEC) onto Ti surfaces [177]. Specifically, the LbL technique takes advantage of the positive and negative charges that each polymer acquires in the appropriate pH conditions, depending on the pK_a_ of their functional groups.

**Figure 11:**
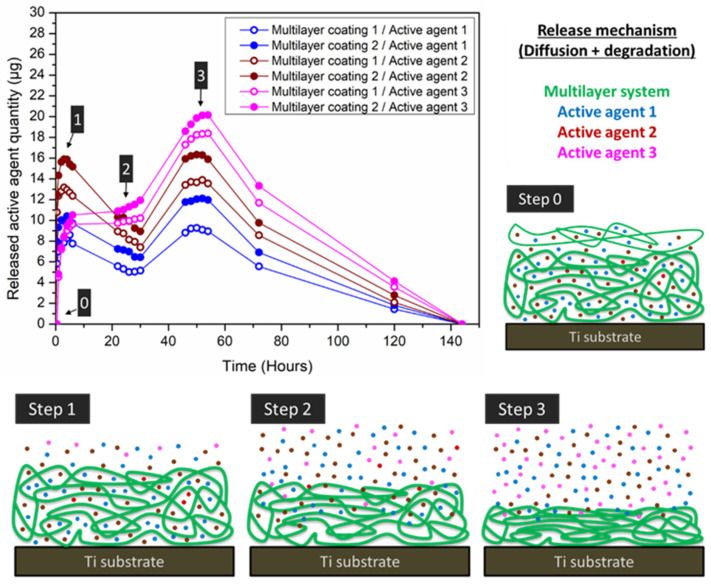
Example of a release-based multilayer coating on Ti surface with cumulative sustained release profile of active agents after 140 h, and the release mechanism from multilayer systems through drug diffusion and multilayer degradation processes.

Although it is true that these multilayered coatings possess lower drug loading capacity than hydrogels, they possess a remarkable control of chemical composition, structure, thickness, homogeneity, and responsiveness [178]. In order to achieve an effective loading and sustained release, either bonds or interactions with intermediate strength are desirable between polymers and the active compound [179]. As in the case of hydrogels, the release of active agents from the multilayer coating takes place typically through diffusion and multilayer degradation processes. Figure 11 illustrates a step-by-step example of the aforementioned release mechanism, in which active agent diffusion and multilayer degradation processes are combined.

Figure 11 illustrates the different stages that can be generally differentiated in the release of sequentially loaded multilayered coatings (steps 1, 2 and 3). A maximum active agent release is usually achieved at 24–48 h due to the general low capacity to retain active agents through the multilayers, except in case of strong and specific drug-polymer interactions, and to the demonstrated low stability of multilayer systems (from hours to few weeks), except in the case of additional crosslinking processes (e.g., hydrogels) [180]. Firstly (step 1), a fast a sharp release occurs due to the degradation of the weakly adhered outer layers evidencing a stage governed by degradation process rather than diffusion process. Then, when active agents are degraded, a second step can be differentiated (step 2). In this stage, release is delayed since active agent degradation is greater than the release. In step 3, almost all the loaded active agents are released by a combination of active agents diffusion and coating degradation [181,182]. The multilayered loading also allows a further release control, because kinetic profiles and released amounts are clearly different according to the position of the loaded layers within the multilayer. This is when the loading of external layers makes an increase of the released amount in comparison to intermediate or inner layers (Figure 9).

In the literature, some remarkable examples of multilayer coatings on Ti-based substrates with potential bioactive properties based on the release of active agents can be found. In 2019, Valverde et al. [183] developed a hyaluronic acid-chitosan multilayer coating onto Ti6Al4V based on electrostatic interactions, which allowed the sustained release of triclosan antibacterial agent. As a result, the release of triclosan from the multilayer coating led to the total loss in bacteria viability demonstrating a powerful bactericidal effect. More recently, Yin et al. [184] presented a polyelectrolyte multilayer coating on the T4-3BL-G Ti implant surface based on chitosan and alginate, able to load interleukin-4 (IL-4) cytokine. In this case, IL-4 sustained release promoted in vitro and in vivo osteogenesis for improved bone formation. Moreover, Wu et al. [185] also investigated a hyaluronic acid-collagen multilayer-coated Ti surface with bone morphogenetic protein-2/7 (BMP-2/7) loading. In this case, osteoblastic differentiation was clearly enhanced after BMP-2/7 release for potential application in bone healing and remodeling. Additional multilayer coatings onto Ti substrates for sustained release of bioactive agents are listed in Table 2.

## 5. Conclusions

Ti and its alloys play a key role as basic materials in orthopedic and dental permanent implants. However, surgery-associated infections as well as the lack of osseointegration can commonly lead to Ti implant failure. To avoid this and to meet the highly demanding requirements of Ti surfaces for this biomedical application, surface modification technologies are still needed. Current and future research is focused on the combination of multiple methods to modify the structure and composition of a wide variety of Ti coatings in order to increase interface bonding and promote active action. This active action includes robust osseointegration and antibacterial properties and has been demonstrated to be successfully implemented by the direct immobilization or the sustained released of the corresponding active agent. Ti surface modification procedures correspond with a sequential layer multi-approach that starts with the inert surface activation inserting –OH reactive sites. The posterior, assembled of organized layers of functionalized silane, catechol or phosphonate derivatives, enables the final linkage of an active layer that ensures the effectiveness of coating. In this review, some of the most employed functionalization approaches in each of the stages of this surface modification sequence reported to date have been summarized.

## Figures and Tables

**Table 1:** Hydrogel coatings for active agents release in biomedical area to create Ti active surfaces.

Hydrogel Coating	Released Active Agent (s)	Biomedical Application	Reference
Polycarboxylic/amino functionalized hyaluronic acid	Vancomycin	Prevention of bacterial adhesion	[154]
Hyaluronic acid	Vancomycin	Enhancement of osseointegration	[155]
	Recombinant human bone morphogenetic protein (rhBMG)-2	Enhancement of peri-implant osteogenesis	[156]
Hyaluronic acid and polylactic acid	Vancomycin Gentamicin Amikacin Tobramycin N-acetylcysteine Sodium salicylate	Enhancement of antibacterial properties	[157]
	Vancomycin Tobramycin	Enhancement of antibacterial properties	[158]
Carboxymethyl chitosan	Silver nanoparticles	Improve antibacterial and bioactive properties	[159]
Carboxymethyl chitosan and chitosan	Interleukin-4 (IL-4) and interferon-γ (IFN-γ) cytokines	Immunomodulation and anti-inflammatory properties	[160]
Chitosan	Vancomycin	Bone regeneration	[161]
	Ibuprofen	Drug elution on conductive implants	[162]
	Ibuprofen	Controlled drug delivery system	[163]
	Interleukin-4 (IL-4) and heparin	Anti-inflammatory, anti-coagulation and anti-thrombus	[164]
	Silver nanoparticles and naproxen	Enhancement of antibacterial and anti-inflammatory properties	[165]
Chitosan and silica xerogel	Fibroblast growth factor	Bioactivity enhancement	[166]
Chitosan and gelatin	Ampicillin	Tissue engineering	[167]
Gelatin	Antimicrobial peptide (AMP) and silicate nanoparticles	Prevention of infections and promotion of bone formation	[168]
Gelatin and alginate	Vancomycin Gentamicin	Reduction of implant-related infection	[169]
Alginate	Dopamine	Regulation of osteoclastic and osteogenic responses	[170]
Alginate and 4-vynilphenylboronic acid	Vascular endothelial growth factor (VEGF)	Local drug delivery system	[171]
Starch	Vancomycin	Prevention of bone infections	[172]
Polyvinyl alcohol (PVA) and phospholipid polymer (PMBV)	Paclitaxel	Anticancer therapy	[173]
poly(2-hydroxyethyl methacrylate)	Ciprofloxacin	Prevent implant associated infections	[174]
poly(ethylene–glycol diacrylate) and acrylic acid	Silver nanoparticles	Enhancement of antibacterial properties	[175]

**Table 2:** Multilayer coatings for active agents release in biomedical area to create Ti active surfaces.

Multilayer Coating	Released Active Agent (s)	Biomedical Application	Reference
Hyaluronic acid and collagen	Enoxacin	Improvement of osteogenesis and osseointegration	[186]
Hyaluronic acid and chitosan	Icariin	Improvement of osteogenesis	[187]
	Silver nanoparticles	Prevention of implant associated infections	[188]
	Antimicrobial peptide-collagen	Long-term sustained antimicrobial activity	[189]
	microRNAs	Enhancement of osteogenic activity	[190]
Hyaluronic acid and polylysine	Parathyroid hormone-related protein (PTHrP)	Enhancement of local bone formation	[191]
Chitosan and bioactive glass	Vancomycin	Prevent implant associated infections	[192]
Chitosan and β-cyclodextrin	Gentamicin	Enhancement of antibacterial properties	[193]
	Calcitriol (VD3)	Promotion of osseointegration	[194]
Chitosan and gelatin	Icariin	Regulation of osteoblast bioactivity	[195]
	Silver nanoparticles	Enhancement of antibacterial properties	[196]
Chitosan and alginate	Minocycline	Enhancement of antibacterial properties	[197]
	Gentamycin	Improvement of bone osseointegration and reduction of bacterial infections	[198]
	Interleukin-4 (IL-4) cytokine	Modulation of macrophage phenotype for tissue repair	[199]
Chitosan, alginate and bovine serum albumin (BSA)	Bone morphogenetic protein-2 (BMP-2)	Tissue engineering	[200]
Dextran and gelatin	A-melanocyte-stimulating hormone (α-MSH)	Improvement of bone remolding	[201]
Polyacrylic acid and poly-L-lysine	Tetracycline	Enhancement of antibacterial properties	[202]
Polyacrylic acid, poly-L-lysine and β-cyclodextrin	Tetracycline	Enhancement of antibacterial properties	[203]
Poly (methacrylic acid) and poly-L-histidine	Bone morphogenetic protein-2 (BMP-2) and fibroblast growth factor (FGF)	Increase of bone growth	[204]

## Data Availability

No new data were created or analyzed in this study. Data sharing is not applicable to this article.
